# Antimicrobial Susceptibility among Urban Wastewater and Wild Shellfish Isolates of Non-O1/Non-O139 *Vibrio cholerae* from La Rance Estuary (Brittany, France)

**DOI:** 10.3389/fmicb.2017.01637

**Published:** 2017-09-12

**Authors:** Sandrine Baron, Emeline Larvor, Séverine Chevalier, Eric Jouy, Isabelle Kempf, Sophie A. Granier, Jean Lesne

**Affiliations:** ^1^Mycoplasmology-Bacteriology Unit, Ploufragan-Plouzané Laboratory, French Agency for Food, Environmental and Occupational Health and Safety (Anses) Ploufragan, France; ^2^Bretagne-Loire University Rennes, France; ^3^Ecole des Hautes Etudes en Santé Publique, Laboratoire d'Etude et de Recherche en Environnement et Santé, Institut de Recherche en Santé, Environnement et Travail, UMR 1085 Rennes, France; ^4^Laboratory for Food Safety, French Agency for Food, Environmental and Occupational Health and Safety (Anses), Paris-Est University Maisons-Alfort, France

**Keywords:** *Vibrio cholerae* non-O1/non-O139, wastewater, wild shellfish, antimicrobial resistance, estuary

## Abstract

The early 2000s marked the end of the Golden age of the antibiotics and the beginning of the awareness on the potential threat to human health due to the dissemination of antimicrobial resistance. As a base-line study, we investigated the antimicrobial susceptibility of 99 strains of non-O1/non-O139 *Vibrio cholerae* isolated from wastewater and shellfish in 2000/2001 within La Rance estuary (Brittany, France). All isolates were susceptible to amoxicillin-clavulanic acid, cefotaxime, imipenem, chloramphenicol, nalidixic acid, ciprofloxacin, norfloxacin, amikacin, gentamicin, tetracycline, doxycycline, trimethoprim-sulfamethoxazole, and erythromycin. The only resistances were to streptomycin, sulfonamides and ampicillin: 54.6% of the isolates had acquired resistance to at least one antimicrobial agent among them and only six isolates from cockles were multidrug resistant. On the basis of the distribution of a limited selection of resistance associated genes, our study shows that *V. cholerae* can constitute an environmental reservoir for these genes. However, none of our isolates harbored integron. This result casts doubt on the capacity of non-O1/non-O139 *V. cholerae* to acquire resistance-associated genes in such context, and on its potential role of indicator of the dissemination of antimicrobial resistance in the aquatic environment.

## Introduction

The early 2000s marked the end of a 50 year time referred as the “Golden age of antibiotics” and the beginning of the awareness of the public health importance of the dissemination of antimicrobial resistance. Recent studies highlighted the important role of the aquatic environment in the dissemination of antimicrobial resistance (Baquero et al., 2008) and particularly wastewater which is considered as a hot spot for horizontal genes transfer (Bouki et al., 2013; Rizzo et al., 2013).

*Vibrio cholerae* is known to be an autochthonous inhabitant of riverine and estuarine aquatic environments and also a waterborne bacterial pathogen. The agent of cholera, a major public health threat for developing countries, belongs to serogroups O1 or O139 and produces the cholera toxin. The other serogroups are collectively referred as non-O1/non-O139 *V. cholerae*. Majority of strains isolated from the environment do not produce the cholera toxin and belong to non-O1/non-O139 serogroups.

In Europe, the increase of the frequency of infections due to non-O1/non-O139 *V. cholerae* in connection with the aquatic environment is reported by numerous authors (Dalsgaard et al., 2000; Ninin et al., 2000; Andersson and Ekdahl, 2006; Ottaviani et al., 2011; Hirk et al., 2016). This increase may be partially related to global warming. Due to the impact of climate change, a rise in the occurrence of *V. vulnificus* and *V. cholerae* is predicted for European waters (Baker-Austin et al., 2017).

*Vibrio cholerae* may act as an environmental reservoir for antibiotic resistance genes (Ceccarelli et al., 2016). It has been established that *V. cholerae*, irrespective of serogroup, has a plastic genome and a long history of successful association with plasmids (Coppo et al., 1995; Carraro et al., 2016). In other words, *V. cholerae*, due to its genetic characteristics, is possibly capable of acquiring and of exchanging genes through either integrons or integrative and conjugative elements (ICE) such as the SXT element (Waldor et al., 1996; Hochhut et al., 2001).

Within La Rance estuary, a survey on the presence of non-O1/non-O139 *V. cholerae* was performed during 2000/2001 in a field of wild shellfish—a point of exposure for humans—and upstream in the discharge of wastewaters from an agglomeration. This estuary opens into The English Channel and is located in Brittany (France). It is a typical environmental aquatic composite, characterized by high anthropogenic pressure. The water body is subject (i) to an artificial tide controlled by tidal power plant, which also causes important sedimentation in the upper part of the estuary, and (ii) to heavy inputs from human activity, mainly diffuse pollution and wastewater discharge from the treatment plant of the Dinan urban area (11,000 inhabitants). It is also an area of intensive recreational activity (sailing, fishing, bathing, collection of shellfish from the shore), and aquaculture (algae cultivation, oyster farming). The estuary is therefore simultaneously an environment receiving discharges from human activities, a potential reactor for genetic exchanges between bacteria subjected to antibiotic pressure and a source of diverse human exposures.

The aim of this study therefore was to investigate the antibiotic susceptibility of isolates of non-O1/non-O139 *V. cholerae* collected in 2000/2001 in cockles and in treated wastewater, in order to be used as base-line study of antimicrobial resistance.

## Materials and methods

### Sampling site and study period

Two sites located in La Rance estuary (North Brittany, France) were sampled in 2000 and 2001. A wild shellfish harvesting area located in Mordreuc (48°30′43 N, 1°58′37 W) on the shore was sampled seven times between June and October 2000 and twice in September 2001.

**Figure 1:**
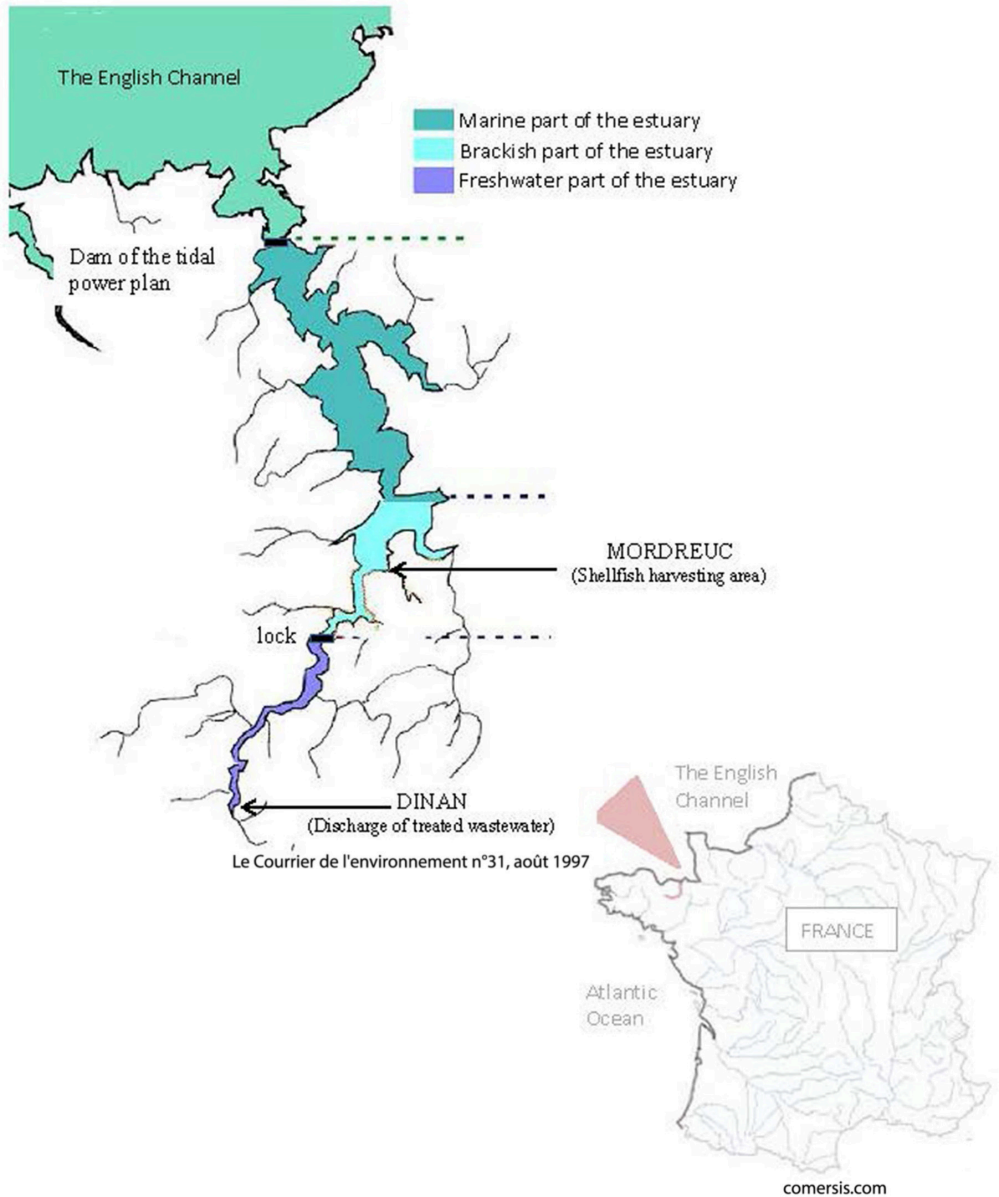
Map of Rance estuary.

Treated wastewater was collected upstream at the outlet of the Dinan biological aerobic treatment plant (BTP) with trickling filters and activated sludge (48°27′48 N, 02°1′40 W). Eleven samples were collected, one every two weeks between June and October 2000 and two more in 2001 (in August and in September). The two sampling sites are 8.5 km apart following the bed of the river (4.8 km between the discharge of wastewater upstream and the lock on the river and 3.7 km between the lock and the wild cockle shellfish harvesting area downstream) (Figure 1).

### Environmental data

For each wastewater sample, salinity, pH and temperature were recorded *in situ*.

The enumeration of *E. coli* and intestinal *Enterococci* was carried out using the 96-wells microplate (BioRad, France) most probable number (MPN) methods ISO 9308-2 and ISO 7899-1, respectively. The detection of *E. coli* in the 96-wells microplate is based on the expression of β-D-glucuronidase enzyme, while β-glucosidase is the target for intestinal *Enterococci*.

### Sample processing

Harvested cockles and water samples were transported to the laboratory in coolers and examined within 3 h of collection.

Upon arrival, wild cockles (*Cerastoderma edule*) were scrubbed under running tap water and opened aseptically, using a sterile knife. 250 g of flesh plus intra-valve liquid were homogenized with a blender in Phosphate-Buffered Saline (1/3 w/w) for two times 30 s. Volumes of 10 mL and 1 mL of homogenate of cockles, and also volumes of 1 mL of tenfold dilutions were mixed with Alkaline Peptone Saline Water (ASPW; composition for 1 liter: 10 g peptone, 20 g NaCl and 5 g yeast extract; post-autoclave pH: 8.6 ± 0.2), in 100 and 10 mL volumes for the raw homogenate, and in 10 mL volumes for the tenfold dilutions.

Sample volumes of 1 L and 100 mL of treated wastewater were filtered (Diaphragm pump N035.3 AN.18 KNF Neuberger, Village-Neuf, France) successively with glass microfiber filters GF/D (grade D, 2.7 μm; Whatman, Maidstone, UK), glass microfiber filters GF/C (grade C, 1.2 μm; Whatman, Maidstone, UK), 0.45 μm cellulose ester membranes (Millipore, Watford, UK) and 0.22 μm cellulose ester membranes (Millipore, Watford, UK). The various filter sizes guaranteed the isolation of both fixed-form and free-living bacteria. For volumes of 1 L and 100 mL, the filters were placed in 250 mL of sterile ASPW. The volumes of 10 mL were incorporated directly in 100 mL of ASPW, and sample volumes of 1 mL to 0.001 mL were incorporated in 10 mL of ASPW.

### Isolation of *V. cholerae*

After incubation at 41 ± 1°C for 16 to 18 h, 0.1 mL of tenfold dilutions of the enrichment were spread over TCBS Agar (Difco) (Muic, 1990) and incubated at 37°C for 36 h. From each enrichment, up to 32 yellow colonies isolated on TCBS Agar were transferred with sterile toothpicks onto Nutrient Agar without NaCl (NA_0_, Difco) to test for growth at 37°C, and then submitted for an oxidase test (Bactident oxidase strips, Merck, Darmstadt, Germany). Positive isolates were considered to be presumptive *V. cholerae* (Baron et al., 2007). Identification of presumptive *V. cholerae* was confirmed by PCR (Nandi et al., 2000). Confirmed isolates (*n* = 216) were maintained at −80°C in brain heart infusion broth (Bio-Rad) containing glycerol (10%). Only one positive isolate per enrichment was conserved for further study (characterization and antibiotic susceptibility).

### Characterization of *V. cholerae*

*Vibrio cholerae* isolates were examined to determine whether they were members of the O1 serogroup via slide agglutination using a polyclonal antibody specific for the O1 surface antigen (Bio-Rad). A saline solution was used as a control to identify self-agglutinating isolates. The genes coding for the O1 and O139 surface antigens (*rfb*) were assessed with PCR using O1-and O139-specific primers (Hoshino et al., 1998).

The virulence associated genes *tcpA, ctxA* and *hlyA* were detected by PCR according to Rivera et al. (2001) and Fields et al. (1992) in the whole collection of confirmed *V. cholerae* non-O1/non-O139. *Vibrio cholerae* O1, Classical and Inaba obtained from Centre National de Référence des Vibrions et du Choléra, Institut Pasteur, Paris (CNRVC 940147), and *Vibrio cholerae* O1, El Tor and Inaba (ATCC 39315) obtained from the collection of the Pasteur Institute (Paris) were included as positive and negative controls where appropriate. All primer pairs, annealing temperatures, and amplicon sizes corresponding to target genes are listed in Table 1.

**Table 1:** The list of primer pairs, annealing temperatures, and amplicon sizes corresponding to target genes for PCR.

**Targeted gene**	**Primer name**	**Primer sequence (5′ - 3′)**	**T°C^a^**	**Amplicon size (bp)**	**References**
*ermA*	ermA1	TAACATCAGTACGGATATTG	54	139	Di Cesare et al., 2013
	ermA2	AGTCTACACTTGGCTTAGG			
*ermB*	ermB1	CCGAACACTAGGGTTGCTC		200	
	ermB2	ATCTGGAACATCTGTGGTATG			
*mef*	mef1	AGTATCATTAATCACTAGTGC		348	
	mef2	TTCTTCTGGTACTAAAAGTGG			
*sul1*	sul1-1	CGCACCGGAAACATCGCTGCAC	65	162	Pei et al., 2006
	sul1-2	TGAAGTTCCGCCGCAAGGCTCG			
*sul2*	sul2-1	TCCGGTGGAGGCCGGTATCTGG	57.5	190	
	sul2-2	CGGGAATGCCATCTGCCTTGAG			
O139 *rfb*	O139-1	AGCCTCTTTATTACGGGTGG	55	449	Hoshino et al., 1998
	O139-2	GTCAAACCCGATCGTAAAGG			
O1 *rfb*	O1-1	GTTTCACTGAACAGATGGG		192	
	O1-2	GGTCATCTGTAAGTACAAC			
*ctxA*	ctx Ats	CTCAGACGGGATTTGTTAGGCACG	64	301	Nandi et al., 2000
	ctx A	TCTATCTCTGTAGCCCCTATTACG			
*ompW*	ompW ts	CACCAAGAAGGTGACTTTATTGTG		588	
	ompW ta	GAACTTATAACCACCCGCG			
*strA*	strA-F	GAGAGCGTGACCGCCTCATT	57	862	Popowska et al., 2012
	strA-R	TCTGCTTCATCTGGCGCTGC			
*strB*	strB-F	GCTCGGTCGTGAGAACAATC	54	859	
	strB-R	AGAATGCGTCCGCCATCTGT			
*ctxA*	94F	AGAATGCGTAAGCCATCTGT	60	564	Fields et al., 1992
	614R	GAATGCGTAAGCCATCTGTTT			
*tcpA* Classical	72F	TCTGCTTCATCTGGCGCTGC	60	620	Rivera et al., 2001
	647R	TCTGCTTCATCTGGCGCTGC			
*tcpA* El Tor	72F	TCTGCTTCATCTGGCGCTGC		451	
	477R	TAACTGCTTCATCTGGCGCTGC			
hlyA Classical	744F	CACAAGGTGACTTTATTGTG		727	
	1184R	CACCAAGAAGGTGACTTTA			
*hlyA* El Tor	489F	TTGTTACCCTTTAATTGGCCC		738/727	
	1184R	CACCAAGAAGGTGACTTTA			

a *Annealing temperature*.

### Enumeration of *V. cholerae*

For samples collected in 2000, enumeration of *V. cholerae* was done by the MPN method (5 tubes, 5 dilutions) based on the number of positive enrichments by serial fractions of volume for a sample (Beliaeff and Mary, 1993). The results were expressed per 100 g of flesh plus intra-valve liquid for cockles and per liter for wastewater. The number of dilutions and tested volumes were adapted according to the expected abundances. In 2001 no enumeration was done, the objective being only to collect isolates.

### Antimicrobial susceptibility testing

The susceptibility of 99 isolates collected from cockles (*n* = 30 in 2000 and *n* = 21 in 2001) and from wastewater (*n* = 31 in 2000 and *n* = 17 in 2001) was studied using the disk diffusion method) according to Clinical and Laboratory Standards Institute (CLSI) guidelines (CLSI, 2015). The 16 antimicrobial agents were chosen in order to represent the main antimicrobial families used in France: ampicillin, amoxicillin-clavulanic acid, cefotaxime, imipenem, chloramphenicol, nalidixic acid, ciprofloxacin, norfloxacin, amikacin, gentamicin, streptomycin, tetracycline, doxycycline, sulfonamides, trimethoprim-sulfamethoxazole association, and erythromycin (Table 1 in Supplementary Material). The CLSI interpretative criteria for disk diffusion susceptibility testing of *Vibrio* spp. (CLSI, 2015) published in the M45 3^rd^ edition were used for ampicillin, amoxicillin-clavulanic acid, cefotaxime, imipenem, chloramphenicol, ciprofloxacin, amikacin, gentamicin, tetracycline, sulfonamides, trimethoprim-sulfamethoxazole association (Table 2). For nalidixic acid, norfloxacin, amikacin and streptomycin, the interpretative criteria for *Enterobacteriaceae* were used (CLSI, 2016) (Table 1 in Supplementary Material). No criterion was available for erythromycin or doxycycline; therefore, the distribution of the inhibition diameters was recorded and interpretation was based on obtained distribution plots. The separation between wild-type (microorganisms without acquired resistance mechanisms) and non-wild-type populations (microorganisms with acquired resistance mechanisms) was determined by visual inspection of the diameter distribution (Hombach et al., 2014). The intermediate results were considered as resistant for this study. Wild-type populations were considered as susceptible populations and non-wild-type as resistant populations.

**Table 2:** Phenotypic and genotypic profile of susceptibility in the 99 isolates of non-O1/non-O139 *V. cholerae*.

**N°**	**Sample**	**Date**	***hly*A^a^**	**Phenotypic antimicrobial suceptibility**	**Molecular determinants**^c^						
				**Resistance profile^b^ (CLSI M45 3^rd^ edition)**	***sul*1**	***sul*2**	***str*A**	***str*B**	***erm*A**	***mef*A**	**Integron**
812	Wastewater	06/19/00	ET	*pansusceptible*	+	+	−	+	+	−	−
816	Wastewater	06/19/00	ET	SSS	+	−	+	+	+	−	−
822	Wastewater	06/19/00	ET	*pansusceptible*	+	−	−	−	+	−	−
823	Wastewater	06/19/00	ET	STR	+	+	−	−	+	−	−
844	Wastewater	06/19/00	–	SSS	−	−	−	−	+	−	−
846	Wastewater	06/19/00	ET	SSS	+	−	−	−	+	−	−
1508	Wastewater	07/03/00	ET	SSS	+	+	+	−	+	−	−
1514	Wastewater	07/03/00	ET	SSS	+	−	−	−	+	−	−
1518	Wastewater	07/03/00	ET	STR	+	−	−	−	+	−	−
1530	Wastewater	07/03/00	Cl	STR	+	−	−	−	+	−	−
1534	Wastewater	07/03/00	ET	*pansusceptible*	+	−	−	−	+	−	−
1538	Wastewater	07/03/00	ET	SSS	+	+	−	−	+	−	−
2602	Wastewater	07/17/00	Cl	SSS	−	−	−	−	+	−	−
2606	Wastewater	07/17/00	ET	SSS	+	−	−	−	+	−	−
2610	Wastewater	07/17/00	ET	SSS	+	−	−	−	+	−	−
2622	Wastewater	07/17/00	ET	SSS	+	−	−	−	+	−	−
2626	Wastewater	07/17/00	Cl	SSS	−	−	−	−	+	−	−
5109	Wastewater	08/14/00	ET	SSS-STR	−	+	+	+	+	−	−
5113	Wastewater	08/14/00	ET	SSS-STR	+	−	−	−	+	−	−
5117	Wastewater	08/14/00	–	STR	+	+	+	−	+	−	−
5125	Wastewater	08/14/00	ET	SSS-STR	−	−	−	−	+	−	−
5129	Wastewater	08/14/00	ET	*pansusceptible*	+	−	−	−	+	−	−
5141	Wastewater	08/14/00	ET	*pansusceptible*	+	−	−	−	+	−	−
5145	Wastewater	08/14/00	Cl	*pansusceptible*	+	−	−	−	+	−	−
6525	Cockles	08/21/00	–	*pansusceptible*	+	+	−	+	+	−	−
6720	Cockles	08/24/00	Cl	SSS	+	−	−	−	+	−	−
6724	Cockles	08/24/00	–	SSS-STR-AMP	+	+	+	−	+	−	−
6728	Cockles	08/24/00	Cl	SSS-STR	+	−	−	−	+	−	−
6732	Cockles	08/24/00	–	*pansusceptible*	+	+	−	+	+	−	−
7101	Wastewater	08/28/00	Cl	SSS	+	−	−	−	+	−	−
7105	Wastewater	08/28/00	ET	*pansusceptible*	+	+	−	+	−	−	−
7113	Wastewater	08/28/00	ET	SSS	+	−	−	−	+	−	−
7133	Wastewater	08/28/00	–	SSS	+	+	−	+	−	−	−
7165	Wastewater	08/28/00	–	*pansusceptible*	+	−	−	−	+	−	−
7169	Wastewater	08/28/00	Cl	*pansusceptible*	−	−	−	−	+	−	−
7173	Wastewater	08/28/00	ET	SSS	−	−	−	−	+	−	−
8621	Cockles	09/04/00	–	SSS-STR-AMP	−	+	−	+	+	−	−
8625	Cockles	09/04/00	ET	SSS	+	−	−	−	+	−	−
8637	Cockles	09/04/00	–	SSS-STR-AMP	+	+	−	+	+	−	−
8645	Cockles	09/04/00	Cl	SSS-STR	+	−	−	+	+	−	−
8660	Cockles	09/04/00	–	SSS	+	−	−	−	+	−	−
9050	Cockles	09/10/00	ET	SSS-STR-AMP	+	−	−	−	+	−	−
9067	Cockles	09/10/00	Cl	*pansusceptible*	+	−	−	+	+	−	−
9083	Cockles	09/10/00	–	AMP	+	−	−	−	+	−	−
9092	Cockles	09/10/00	–	*pansusceptible*	+	−	−	−	+	−	−
9112	Cockles	09/10/00	ET	SSS	+	−	−	+	+	−	−
9114	Cockles	09/10/00	–	SSS	+	+	−	+	+	−	−
10205	Cockles	09/18/00	ET	SSS-AMP	+	−	−	−	+	−	−
10225	Cockles	09/18/00	ET	SSS	+	−	−	−	+	−	−
10229	Cockles	09/18/00	–	SSS-STR-AMP	+	−	−	−	+	−	−
10257	Cockles	09/18/00	ET	SSS-STR	+	+	+	+	+	−	−
10285	Cockles	09/18/00	–	SSS	+	−	−	−	+	−	−
11013	Cockles	09/24/00	ET	SSS-STR-AMP	−	−	−	−	+	−	−
11017	Cockles	09/24/00	–	SSS	−	−	−	−	+	−	−
11021	Cockles	09/24/00	–	SSS	+	−	−	−	+	−	−
11037	Cockles	09/24/00	ET	SSS	+	−	−	−	+	−	−
11045	Cockles	09/24/00	–	SSS	+	−	−	−	+	−	−
11053	Cockles	09/24/00	Cl	SSS	+	−	−	−	+	−	−
11057	Cockles	09/24/00	ET	STR	+	−	−	−	+	−	−
11063	Cockles	09/24/00	–	*pansusceptible*	+	−	−	−	+	−	−
11109	Cockles	09/24/00	–	SSS-AMP	+	+	−	+	+	−	−
21850	Wastewater	08/06/01	Cl	*pansusceptible*	+	+	−	−	+	−	−
21852	Wastewater	08/06/01	–	*pansusceptible*	−	−	−	−	+	−	−
21853	Wastewater	08/06/01	–	*pansusceptible*	+	−	−	−	+	−	−
21854	Wastewater	08/06/01	–	*pansusceptible*	+	+	−	−	+	+	−
21855	Wastewater	08/06/01	–	STR	+	−	−	−	+	−	−
21856	Wastewater	08/06/01	ET	*pansusceptible*	+	+	−	−	+	+	−
21857	Wastewater	08/06/01	–	*pansusceptible*	+	+	−	−	+	−	−
21858	Wastewater	08/06/01	–	*pansusceptible*	+	+	−	−	+	−	−
21859	Wastewater	08/06/01	–	*pansusceptible*	+	−	−	−	+	−	−
21860	Wastewater	08/06/01	–	*pansusceptible*	+	+	−	−	+	−	−
21861	Wastewater	09/18/01	–	STR	+	+	−	−	+	+	−
21863	Wastewater	08/06/01	–	*pansusceptible*	+	+	−	−	+	+	−
21864	Wastewater	08/06/01	ET	SSS	+	−	−	−	+	−	−
21865	Wastewater	08/06/01	Cl	*pansusceptible*	+	+	−	−	+	+	−
22410	Wastewater	08/20/01	–	*pansusceptible*	+	+	−	−	+	−	−
22415	Wastewater	08/20/01	ET	SSS	+	+	−	−	+	−	−
22416	Wastewater	08/20/01	–	*pansusceptible*	+	+	−	−	+	−	−
28202	Cockles	09/18/01	Cl	*pansusceptible*	+	+	−	−	+	+	−
28203	Cockles	09/18/01	Cl	STR	+	+	−	−	+	+	−
28204	Cockles	09/18/01	–	*pansusceptible*	+	+	−	−	+	−	−
28205	Cockles	09/18/01	–	*pansusceptible*	+	+	−	−	+	+	−
28206	Cockles	09/18/01	Cl	*pansusceptible*	+	+	−	−	+	−	−
28207	Cockles	09/18/01	–	*pansusceptible*	+	+	−	−	+	−	−
28208	Cockles	09/18/01	–	*pansusceptible*	+	+	−	−	+	+	−
28209	Cockles	09/18/01	ET	*pansusceptible*	+	+	−	−	+	−	−
28210	Cockles	09/18/01	–	*pansusceptible*	+	+	−	−	+	+	−
28211	Cockles	09/18/01	Cl	STR	+	+	−	−	+	+	−
28219	Cockles	09/18/01	ET	*pansusceptible*	+	+	−	−	+	−	−
28220	Cockles	09/18/01	ET	*pansusceptible*	+	+	−	−	+	−	−
28222	Cockles	09/18/01	–	*pansusceptible*	−	+	−	−	+	−	−
28223	Cockles	09/18/01	–	*pansusceptible*	+	+	−	−	+	−	−
28224	Cockles	09/18/01	Cl	STR	−	+	−	−	+	+	−
28229	Cockles	09/18/01	–	*pansusceptible*	+	+	−	−	+	−	−
28237	Cockles	09/18/01	Cl	*pansusceptible*	+	+	−	−	+	+	−
28238	Cockles	09/18/01	Cl	*pansusceptible*	+	+	−	−	+	+	−
28242	Cockles	09/18/01	–	*pansusceptible*	+	+	−	−	+	−	−
28253	Cockles	09/18/01	Cl	*pansusceptible*	+	+	−	−	+	−	−
28254	Cockles	09/18/01	Cl	*pansusceptible*	+	+	−	−	+	−	−

a *ET, hemolysin El Tor; Cl, hemolysin Classical; −, no hemolysin detected*.

b *Resistance profiles were defined using with CLSI breakpoint when available in other cases referred to Table 1 in Supplementary Material*.

c *+, gene or integron was detected by PCR; −, gene or integron was not detected by PCR*.

*Pansusceptible, profile susceptible to all the tested antimicrobial agents*.

*SSS, sulfonamide; STR, streptomycin; AMP, ampicillin*.

### Antibiotic resistance associated genes

Screening for resistance-associated genes in our collection of isolates was done particularly to provide information on the reservoir of resistance genes in the autochthonous aquatic non-O1/non-O139 *V. cholerae* populations under study. As, in the early 2000s CTX-M enzymes have become the most prevalent extended-spectrum β-lactamases in Europe (Cantón and Coque, 2006), the *bla*_CTX−M_ was detected using PCR (Woodford et al., 2005). Genes associated with resistance to streptomycin (*strA* and *strB*), to sulfonamides (*sul1* and *sul2*)—which may be associated with the presence of *V. cholerae* SXT element—and to erythromycin (*ermA, ermB* and *mef*) were detected by PCR (Pei et al., 2006; Popowska et al., 2012; Di Cesare et al., 2013). Class 1, 2, and 3 integrons were screened using qPCR (Barraud et al., 2010). All primer pairs, annealing temperatures and amplicon sizes corresponding to target genes are listed in Table 1.

### Statistical analysis

To compare the characteristics of the isolates collected from cockles and wastewater the chi-squared test, and Pearson's chi-squared test whenever needed, was used.

## Results

### Characterization and enumeration of *Vibrio cholerae*

None of the 216 isolates (cockles: 116, wastewater: 100) characterized for the MPN estimation—one isolate by positive enrichment broth—belonged to the O1 or the O139 serogroup (No agglutination was observed; absence of the *rfb* sequence gene), and carried neither the *ctxA* gene, nor the El Tor or Classical variants of the *tcpA* gene. All *V. cholerae* isolates were therefore non toxigenic.

The frequency of the hemolysin gene variants El Tor or Classical was 52.3% (113 strains), without any difference between wastewater and cockle isolates (59.0 vs. 46.5%). The El Tor variant was detected in 66 isolates (30.5%) and the Classical variant in 47 isolates (21.8%).

**Figure 2:**
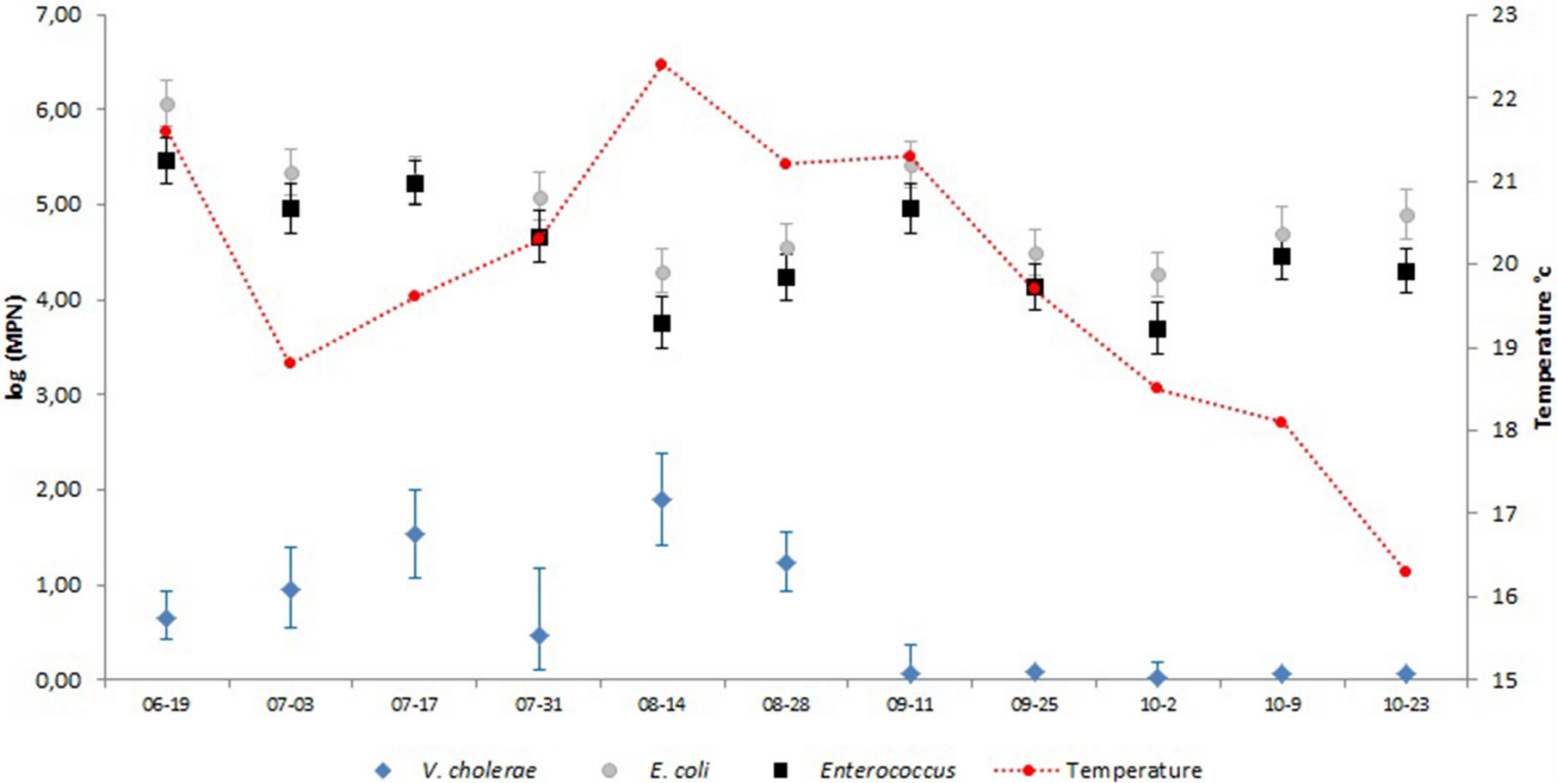
Densities of *V. cholerae, E. coli* and intestinal *Enterococci* in treated wastewater. Densities of *E. coli* and intestinal *Enterococci* were expressed by 100 mL of water and *V. cholerae* per 1 liter of water.

In 2000, *V. cholerae* densities were recorded in cockles and in treated wastewater. In cockles, between June and September, concentrations of *V. cholerae* ranged from 2.5 to 230 MPN/100 g of flesh plus intra-valve liquid. The highest density was observed in September and *V. cholerae* was detected in all seven samples (Table 3). At the outlet of the biological aerobic treatment plan, *V. cholerae* was detected in 75% of the samples (9/12), and densities ranged from 0.075 MPN/L to 78.2 MPN/L (Figure 2). Abundance of *V. cholerae* was below the detection limit of the method once in September (09/25/2000–detection limit: 0.198 MPN/L) and twice in October (10/09/2000 and 10/23/2000—detection limit: 0.181 MPN/L). The highest densities were observed in August. The geographic mean is 0.6 log_10_ (MPN/L). The temperature followed the seasonal variation and ranged from 22.2°C in August to 16°C in October (Figure 2). The pH ranged from 6.31 to 7.51 with an average of 7.40 ± 0.43. The salinity ranged from 0.2 to 0.9‰. The densities of *E. coli* and intestinal *Enterococci* were relatively stable during the sampling period with geometric mean value of 4.9 × 10^5^ ± 0.5 log_10_ (MPN/100 mL) and 4.5 ± 0.6 log_10_ (MPN/100 mL) respectively. Means densities per liter of *E. coli* were 5.3 log_10_ unit higher than *V. cholerae* ones and intestinal *Enterococci* were 4.8 log_10_ unit higher than *V. cholerae* densities (Figure 2).

**Table 3:** Densities of *V. cholerae* in cockle samples (MPN per 100 g of flesh plus intra-valve liquid) according to sampling dates.

**Sampling dates (month/day)**	**08/24/2000**	**09/04/2000**	**09/17/2000**	**09/18/2000**	**09/24/2000**	**10/02/2000**	**10/16/2000**
MPN	3.8 × 10^1^	2.3 × 10^2^	1.0 × 10^2^	3.4 × 10^2^	8.0 × 10^1^	2.5 × 10^0^	1.5 × 10^2^

In 2001, sampling dates were chosen in August and September to optimize the collection of isolates from cockles and wastewater.

### Antimicrobial susceptibility

All the 99 isolates studied were susceptible to the 11 following antimicrobial agents: amoxicillin-clavulanic acid, cefotaxime, imipenem, chloramphenicol, nalidixic acid, ciprofloxacin, norfloxacin, amikacin, gentamicin, tetracycline, trimethoprim-sulfamethoxazole association (Table 2).

For doxycycline and erythromycin, no breakpoint were available, based on the distribution of the diameter of inhibition (Figures 4A,B), only one population could be observed. All the strains were considered as susceptible (Baron et al., 2016).

Among the 54 isolates (54.6%) that were resistant to at least one antimicrobial agent, 28 were collected in cockles and 26 in wastewater. The most frequent resistance was to sulfonamides (44 isolates), followed by streptomycin (22 isolates) and ampicillin (9 isolates). No significant difference was observed for the frequency of resistance to either sulfonamides or streptomycin between the isolates from cockles and those from wastewater. The nine isolates resistant to ampicillin were collected from cockles in 2000.

**Figure 3:**
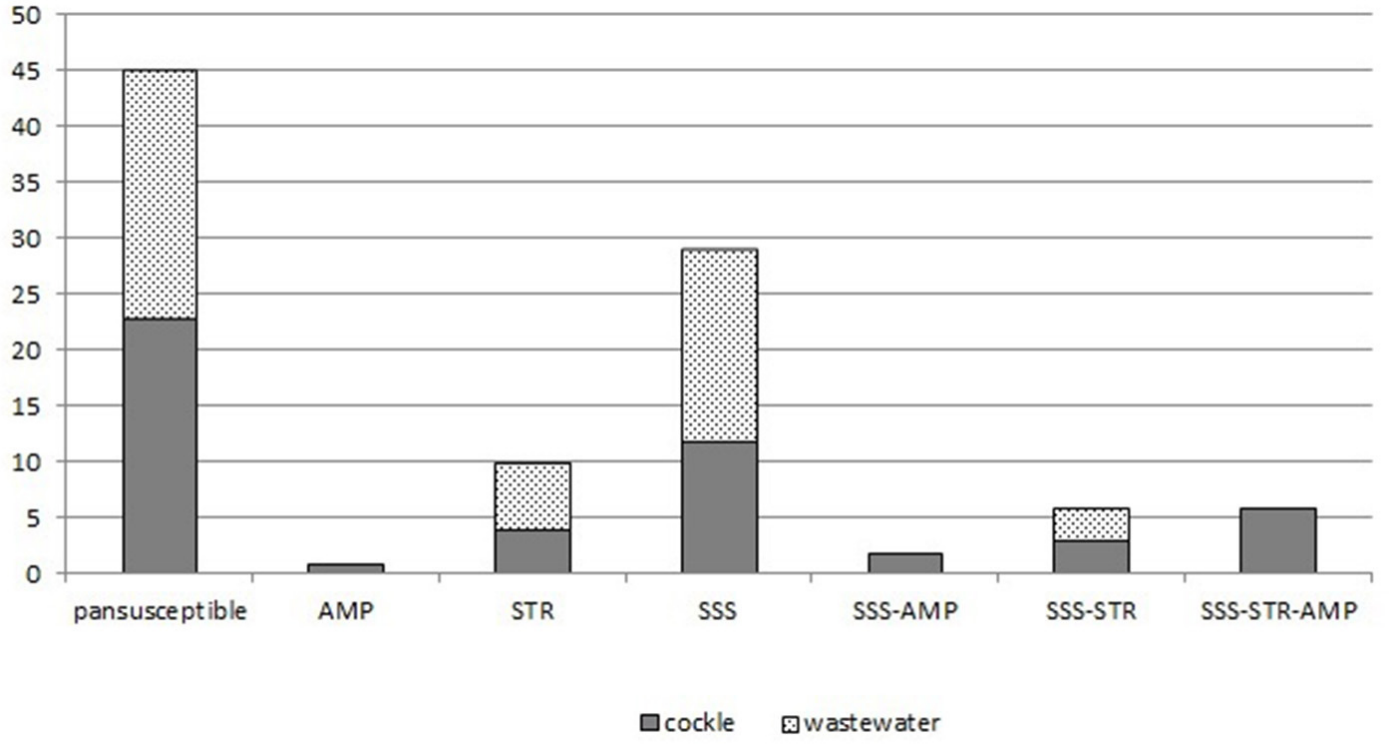
Distribution of resistance profiles among the 99 isolates of non-O1/non-O139 *Vibrio cholerae*.

The most frequent resistance profile was susceptibility to the 16 antimicrobial agents tested (*n* = 45), then resistance to sulfonamides only (*n* = 29), followed by resistance to streptomycin only (*n* = 10). Six multi-drug resistant isolates were detected in cockles in 2000; none in wastewater. They were resistant to sulfonamides, ampicillin and streptomycin (Figure 3).

**Figure 4:**
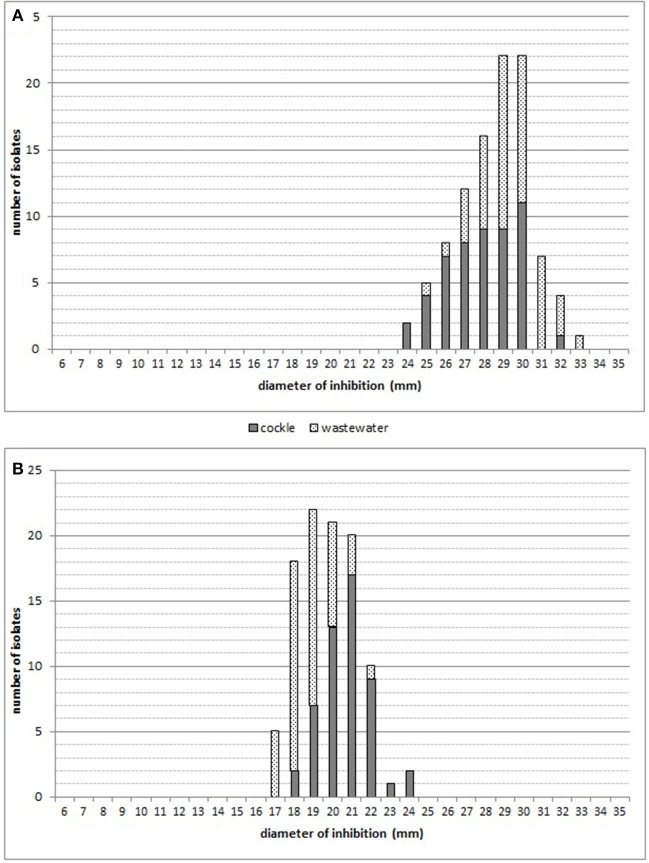
Distribution of diffusion zone diameters: **(A)** doxycycline (30 μg); **(B)** erythromycin (15 μg).

No difference was observed in the frequency of associated resistance genes between cockles and wastewater. Among the 99 isolates, neither the *erm*B nor the *bla*_CTX−M_ gene was detected. The most frequent gene was *erm*A (*n* = 97); *sul1* and *sul2* were detected in 86 and 49 isolates respectively (Table 4). None of the three classes of integron were detected in the 99 *V. cholerae* strains (Table 2).

**Table 4:** Distribution of resistance-associated genes among the 99 isolates of non-O1/non-O139 *Vibrio cholerae*.

	**Cockle**	**Wastewater**	**Total**
*erm*A	2	7	9
*sul*1 *erm*A	17	20	37
*sul*2 *erm*A	1		1
*sul*1 *sul*2 *erm*A	11	9	20
*sul*1 *str*B *erm*A	3		3
*sul*1 *sul*2 *str*B		2	2
*sul*2 *erm*A *mef*	1		1
*sul*2 *str*B *erm*A	1		1
*sul*1 *sul*2 *erm*A *mef*	8	5	13
*sul*1 *sul*2 *str*B *erm*A	5	1	6
*sul*2 *str*A *str*B *erm*A		1	1
*sul*1 *str*A *str*B *erm*A		1	1
*sul*1 *sul*2 *str*A *erm*A	1	2	3
*sul*1 s*ul*2 *str*A *str*B *erm*A	1		1
Total	51	48	99

Among the 45 isolates harboring *sul1* and *sul2*, 10 were resistant to sulfonamides and 35 were susceptible. Similarly, for the isolates harboring *str*A and *str*B, two were resistant and four were susceptible to streptomycin. All the isolates harbored at least one of the eight targeted genes and 52.3% of the isolates harbored at least three genes. Only two isolates harbored simultaneously *sul*2, *str*A and *str*B (Table 4).

## Discussion

The objective of this baseline study was to characterize the susceptibility to antibiotics of non-toxigenic *V. cholerae* collected in 2000/2001 in a cholera-free area, at two points of interface between the aquatic environment and the human population—a discharge of urban wastewater and shellfish harvested from the shore downstream.

### Abundance of *Vibrio cholerae*

The presence of *V. cholerae* in treated wastewater had never been described in France. Non-O1/non-O139 *V. cholerae* was detected in 75% of the wastewater samples at the outlet of the treatment plant of the city of Dinan. An investigation from our team conducted at the same time in stabilization ponds at Saint-Helen, a village in the watershed of La Rance estuary, confirmed the persistent presence of *V. cholerae* in wastewater at another location (data not shown). To our knowledge, it can only be compared in Europe to the results of an Italian study: 19 strains were isolated from 69 samples of wastewater at the inlet and the outlet of a treatment plant (Gatti et al., 1997).

The levels of abundance of non-O1/non-O139 *V. cholerae* observed in the treated wastewater from Dinan (0.075 MPN/L to 78 × 10^1^ MPN/L) were much lower than those calculated with a comparable method of analysis, outside the context of cholera, in Mediterranean Europe (Yugoslavia, wastewater discharge of a coastal town with 600,000 inhabitants, 1990: range from 2.0 × 10^2^ to 2.1 × 10^5^ MPN/L) (Muic, 1990), or in North Africa (Marrakesh, at the outlet of experimental stabilization ponds, from January 1986 to December 1987: range from < 3 × 10^2^ to 2.3 × 10^7^ MPN/L (Lesne et al., 1991) and from October 1992 to September 1993: geometric mean in winter of 2.5 × 10^4^ MPN/L, and geometric mean in summer of 1.7 × 10^6^ MPN/L (Mezrioui and Oufdou, 1996). The only other recent study, to our knowledge, was done in South Africa from August 2011 to May 2012 (17 dates) but in the context of a cholera epidemic (Okoh et al., 2015). The levels of abundance of *V. cholerae* observed in four wastewater treatment plants discharging into rivers at four sampling points each (influent, effluent, upstream, downstream), i.e., in 272 samples of water, were still higher (1.6 × 10^3^ CFU/L to 6.3 × 10^9^ CFU/L), but the method of analysis in that study is not comparable to ours. And an unspecified fraction of the counted population of *V. cholerae* is toxigenic in 40% of the samples, which is not the case in any of the other studies presented.

Sewage is oligohaline water, rich in organic matter, with a pH neutral to alkaline, characteristics which meet the requirements of *V. cholerae* for growth or survival. The abundance of *V. cholerae* also appeared higher under climates that include a hot season, which can be considered alongside the well-known favorable effect (direct or indirect) of water temperature on the growth of population of this bacterium in aquatic ecosystems. While it is well established that *V. cholerae* is autochthonous of the natural aquatic environment, its detection in sewage fed by a public water distribution network raises the question of its origin. At least two non-mutually-exclusive assumptions can be made: contribution of run-off waters and contribution of a possible healthy carriage in the gut of the human population (Morris, 1990). In any case, it seems that sewage establishes an ecosystem favorable to the installation and development of this species. We have no information about the presence of healthy carriers in the local human population, nor on local clinical cases of infection with non-O1/non-O139 *V. cholerae*. It is interesting to observe that intestinal carriage also exists in livestock (Rhodes et al., 1985) and that in our study, strains of non-O1/non-O139 *V. cholerae* have also been isolated in the wastewater treatment plants of two neighboring slaughterhouses, one for pigs and the other for calves (Sandrine Baron, personal communication).

Between June and September 2000, *V. cholerae* was detected in all seven samples of cockles. The field of naturally-growing cockles was located in brackish water area (salinity between 0.5 and 30‰) in the upper estuary. Similar results were obtained in a field of mussels (*Mytilus edulis*) located in a zone where sea water (Northern Sea) mixes with brackish water (Baltic Sea), in The Sound (between Sweden and Denmark) during summer 2006: 100% (*n* = 19) of samples contained non-toxigenic *V. cholerae* (Collin and Rehnstam-Holm, 2011). In other salinity conditions, such as marine waters (salinity ≈33‰) or polyhaline waters (salinity between 18 and 30‰), the frequencies of detection are much lower. In France, in summer 1999, only two strains of non-O1/non-O139 *V. cholerae* were detected in two samples of mussels from the Channel, and two strains in one sample of mussels from the Atlantic Ocean (Hervio-Heath et al., 2002). Again in France, between 2006 and 2007, *V. cholerae* was not detected in mussels and clams of two lagoons which are open onto the Mediterranean Sea (Cantet et al., 2013). In Italy, during a national study from May to September 2006 on the Ligurian Adriatic and Tyrrhenian coasts, non-O1/non-O139 *V. cholerae* was present in only 7.8% (*n* = 90) of mussel samples and never detected in the clam samples (*n* = 56) (Ottaviani et al., 2009). In 2011–2014, another study detected *V cholerae ctx*^−^*tcp*^−^ in only one sample of clams out of 112 collected (Passalacqua et al., 2016). In Germany, during a study of non-O1/non-O139 *V. cholerae* in mussels produced in the Wadden Sea, the frequencies of presence were 16% (*n* = 46) in 2012 and 11% (*n* = 71) in 2013 (Huehn et al., 2014). On the coast of the Atlantic Ocean, north of São-Paulo (Brazil), in mussels cultivated in sea water, less than 10% of samples collected between February 1989 and February 1990 were positive for non-O1/non-O139 *V. cholerae*. On the Northeast Atlantic Coast in Long Island Sound (USA) *V. cholerae* was detected in 8.8% (*n* = 68) of oyster samples (*Crassostea gigas*) and in 3.3% (*n* = 30) in clams. All these data show a higher frequency of detection of *V. cholerae* in brackish waters than in marine waters.

The following studies are the only ones that supply quantitative data of *V. cholerae* in shellfish. In Brazil, with a method similar to ours, the abundances are lower, varying between 7 and 2.3 × 10^1^ MPN/100 g (Matté et al., 1994). In the USA, by using a PCR multiplex on enrichment broth, the abundances observed were between 3.6 × 10^1^ and 3.0 × 10^3^ MPN/100 g for oysters and 3.0 × 10^2^ in the only sample of positive clams (Jones et al., 2014). This difference of method might explain the higher abundances.

### Characterization of the isolates

In France, the rare cases of cholera are imported (Geneste et al., 2000; Tarantola et al., 2007), thus the presence of the main virulence factors of cholera, *ctxA* and *tcpA*, is very improbable in the aquatic population of *V. cholerae*. The products of the *ctxA* gene are involved in interactions with the host that are responsible for pathological damage (Wassenaar and Gaastra, 2001). This gene encodes the cholera toxin A subunit. The *tcpA* gene is a colonization gene encoding the principal subunit of the toxin-coregulated pilus (type IV), which appears to facilitate the interaction between bacteria and the intestinal epithelium surface (Reidl and Klose, 2002). In our collection, the *ctxA* gene and the *tcpA* gene were never detected. Similarly, none of 11 isolates of non-O1/non-O139 *V. cholerae* collected from seafood samples (Italian coastal waters), harbored either the *ctxA* or the *tcpA* genes (Ottaviani et al., 2009). Also in a collection of 395 strains of non-O1/non-O139 *V. cholerae* collected in Chesapeake Bay, Maryland (USA), in water, sediment and oyster samples during a *Vibrio* surveillance program (2009 to 2012), only four isolates harbored the *ctxA* gene and none the *tcpA* gene (Ceccarelli et al., 2015).

Inversely, *hlyA* genes are frequently harbored by environmental isolates of non-O1/non-O139 *V. cholerae*. The *hlyA* gene encodes hemolysin, the production of which was originally used to differentiate Classical and El Tor *V. cholerae* O1 biotypes. Its role in virulence is not clearly established. However, it does not seem to be an essential factor for cholera-type human pathogenesis, since the hemolytic function is absent in clinical isolates of the 6th pandemic (Classical biotype), and disappears gradually from isolates of the 7th (El Tor biotype) (Barrett and Blake, 1981). By contrast, in non-O1/non-O139 strains associated with cases of gastro-enteritis and diarrhea, but lacking virulence factors associated with cholera, hemolysin production might constitute an important virulence factor. In our study, 52.3% of the *V. cholerae* isolates harbored the hemolysin gene (variant El Tor or Classical without distinction) and no significant difference between cockles and wastewater was noticed. In comparison, all the 13 isolates of *V. cholerae* collected from seafood in Italy harbored *hlyA* (Ottaviani et al., 2009). Among 39 non-O1/non-O139 *V. cholerae* strains from Brazil, 94.9% showed homology to El Tor hemolysin, 2.6% were associated with Classical hemolysin, and 2.5% were negative for both genes (Rivera et al., 2001). Lastly, among the 395 environmental isolates collected by Ceccarelli et al. (2015), 83% were *hlyA* positive, including the seven oyster isolates. In these studies, the El Tor variant was thus either the only variant detected or the most abundant. In our study, the El Tor variant was found in only 30.5% (*n* = 66) of the isolates and the Classical variant in 21.8% (*n* = 47). The Classical biotype has an 11 bp deletion within the *hlyA* coding region, compared to the El Tor biotype, resulting in a truncated and non-hemolytic *hlyA* product which may have another, as yet unknown, function (Rader and Murphy, 1988; Alam et al., 1997). It suggests that a part of our isolates would not be capable of producing a functional hemolysin, or at best a less active one. It could be verified by carrying out *in vitro* tests following the example of those performed by Ottaviani et al. (2009).

Other genes, such as *rtxA, chxA, T6SS, hapA, nanH*, etc…are suspected of being involved in the virulence of toxigenic *V. cholerae*; but this does not mean that their role is well defined. Their detection in environmental isolates helps to better characterize these isolates and to estimate the genetic reservoir associated with virulence, but does not permit any conclusion concerning the pathogenicity of a strain. Indeed, their presence can be interpreted by precaution as enabling the bacterium to induce acute gastroenteritis if present in shellfish (Ceccarelli et al., 2015). But at present, there are still no markers of virulence validated for the pathogenic strains of non-O1/non-O139 *V. cholerae*. In view of the variety of the pathologies caused by non-O1/non-O139 *V. cholerae*, very numerous markers will probably be necessary to cover all the mechanisms of pathogenicity. These data would be essential to better manage the hazard for human health represented by the environmental isolates, in the context of the increase of cases of human vibriosis in Europe (Baker-Austin et al., 2016).

### Antimicrobial susceptibility

The existence of environmental strains of non-O1/non-O139 *V. cholerae* presenting acquired resistances was reported in the literature in cholera-free regions. Therefore, we undertook the study of the antibiotic resistance by choosing two compartments exposed to different pressures of antibiotics: wastewater coming from the sewage system of an urban area containing a hospital, and a field of wild shellfish located downstream in the estuary. This estuary is characterized by a low pressure of ground breeding and a few zones contaminated by farm effluent, but no fish farming.

In case of infection by non-O1/non-O139 *V. cholerae*, ciprofloxacin, or/and doxycycline or extended spectrum cephalosporin are the recommended treatments (Daniels and Shafaie, 2000). As all isolates tested here were susceptible to these antimicrobial agents, the loss of efficacy of such first line treatments was not to be feared.

Among the 99 environmental isolates tested, resistances were observed for only three antimicrobial agents: ampicillin, streptomycin and sulfonamides, and no significant difference was observed between wastewater (*n* = 48) or cockles (*n* = 51) origin These molecules are considered as “old antibiotics.” In a similar context (cholera free area, temperate climate, and labeled use of antibiotics), recent studies on environmental isolates–collected in the Baltic Sea (Germany) (*n* = 131 isolates, between 2008 and 2014) (Bier et al., 2015) and in the Chesapeake Bay (USA) (*n* = 307 isolates, between 2009 and 2014) (Ceccarelli et al., 2015) were performed between 2008 and 2014. In our study, 9.0% of the isolates were resistant to ampicillin, result in agreement with those of the two previous ones (16.3% in the Chesapeake Bay and 7.6% in the Baltic Sea).

The resistance profiles observed in our strains collected in wastewater were very similar to those observed in *V. cholerae* isolated at the outlet of stabilization ponds in Marrakesh 10 years previously, except for streptomycin. The highest observed resistance rates were for streptomycin (11.8%), and ampicillin (9.1%) (Imziln and Hassani, 1994). All the 120 isolates of non-O1/non-O139 *V. cholerae* tested were susceptible to quinolones (nalidixic acid) and phenicols (chloramphenicol) and only one isolate was resistant to trimethoprim-sulfamethoxazole association. More recently, in Tanzania, all the strains of *V. cholerae* collected in wastewater were resistant to ampicillin and to tetracycline but quite susceptible to phenicol or quinolone (Hounmanou et al., 2016).

Wastewater is considered to be hot spots for the acquisition of resistance. Nevertheless, in our study, it is worth noting that there was not more resistance in wastewater than in shellfish. One of the explaining hypotheses could be the relatively low abundance of this bacterium in wastewater, compared to enteric species (like *E. coli* or intestinal *Enterococci*).

Six multidrug resistant strains were collected from cockles; they were resistant to ampicillin, streptomycin and sulfonamides (6% of the isolates). The multi-resistant strains of environmental origin are rare in cholera-free areas. Bier et al. (2015) and Ceccarelli et al. (2015) thus detected no multi-resistant strains among 131 and 307 isolates respectively. But these authors did not test sulfonamides, which in our study accounted for the most frequent resistance.

All our isolates were susceptible to extended spectrum cephalosporins and none harbored the *bla*_CTX−M_ gene, one of the most important genes conferring this resistance to *Enterobacteriaceae* in France. These isolates were collected in 2000 and 2001; the dissemination of this gene in environmental strains appeared later and so its non-detection in our strains was not surprising. Even though 29.5% (62/210) of non-O1/non-O139 *V. cholerae* isolated from tropical seafood in Cochin (India) in 2013 were resistant to cefpodoxime (Kumar and Lalitha, 2013), clearly, extended spectrum β-lactamase enzymes are uncommon in *V. cholerae* (Ceccarelli et al., 2016).

All isolates of *V. cholerae* collected in 2000–2001 in La Rance estuary were also susceptible to carbapenem, another critical antimicrobial agent. Since the beginning of the twenty-first century, environmental *V. cholerae* harboring carbapenemase genes have been reported several times in the literature, but this could be a bias linked to the interest focused on this antimicrobial resistance. Indeed, a gene conferring resistance to carbapenems has been detected in environmental isolates of non-O1/non-O139 *V. cholerae*, even in Europe. In France, one strain of *V. cholerae* isolated from cloacal swab samples from juvenile unfledged yellow-legged gulls (*Larus michahellis*) coharbored the *bla*_VIM−1_ and *bla*_VIM−4_ carbapenemase genes (Aberkane et al., 2015). Bier et al. (2015) identified a non-toxigenic *V. cholerae* harboring a carbapenemase gene that could not be identified by standard PCR typing. In Canada, a novel Amber class A carbapenemase was found in non-toxigenic *V. cholerae* strains isolated from shrimp intended for human consumption (Mangat et al., 2016). Given the potential ability of conjugative plasmids to transfer naturally between enterobacterial populations in the intestinal gut (Rashid and Rahman, 2015), the aquatic environment is now considered as an ideal setting for acquisition and dissemination of antibiotic resistance (Marti et al., 2014) and the horizontal transfer of ESBL/carbapenemase genes to *V. cholerae* cannot be ruled out.

On the basis of the distribution of a limited set of resistance genes (eight), our study shows that *V. cholerae* can constitute an environmental reservoir for these genes. However, none of the 99 isolates studied harbored integron. This result is in agreement with those of Bier et al. (2015) and Ceccarelli et al. (2015). As such mobile genetic elements play an important role in the acquisition and dissemination of antimicrobial resistance; this provides some confirmation for the conclusion drawn above from our susceptibility data, that, in our geographic context at least, non-O1/non-O139 *V. cholerae* seems not to have significant role in the dissemination of antibiotic resistance in the environment.

Nevertheless, integrons are not the only medium by which *V. cholerae* acquires antimicrobial resistance genes. Presence of integrative and conjugative element (ICE) has been regularly reported since the first description of the so-called SXT element in *V. cholerae* (Waldor et al., 1996) confering resistance to sulfonamides, trimethoprim, streptomycin and chloramphenicol. Further study should probably also target detection of specific markers to evaluate the presence of the SXT element in this non-O1/non-O139 *V. cholerae* collection. But, since multiple studies investigating either clinical or non-pathogenic collections of *V. cholerae* (Ceccarelli et al., 2006; Mala et al., 2016, 2017; Wang et al., 2016) had demonstrated that antimicrobial resistance genes carried by the SXT element are various, and *sul2, strA* and *strB* appeared to be the most commonly encountered resistance genes. We might hypothesize that the proportion of strains from our collection harboring the SXT element might be quite low.

## Conclusion

In France, a cholera-free country, the presence of *V. cholerae* non-O1/non-O139 in treated wastewater of an urban area (11,000 inhabitants), and in cockles had never been investigated simultaneously in a same geographic area.

In similar epidemiological contexts, without any history of cholera cases but potentially different local antibiotic pressures, this study and two others (Bier et al., 2015; Ceccarelli et al., 2015) performed later on cast doubt on the capacity of *V. cholerae* non-O1/non-O139 to acquire resistance-associated genes and its potential role as indicator for the dissemination of antimicrobial resistance in the aquatic environment. However, *V. cholerae* presents the advantage of being a bacterium able to develop in the aquatic estuarine environment, unlike enteric bacteria which only survive for a limited time in receiving surface waters. Moreover, *V. cholerae* is known to be susceptible to carbapenems and to third generation cephalosporins, unlike *Aeromonas* spp., which is also more and more often proposed as candidate to be an indicator bacterium of antimicrobial resistance circulating in the aquatic environment (Usui et al., 2016; Varela et al., 2016; Baron et al., 2017). So, detection of *V. cholerae* harboring a mechanism of resistance to antibiotic critically important for Public health, such as carbapenems or third generation cephalosporins, could establish an alert on contaminated ecosystems.

Finally, those historical data would deserve now to be refreshed in order to determine if there has been a shift in the antimicrobial resistance genes harbored by the population of *V. cholerae* non-O1/non-O139 from La Rance Estuary over the past 20 years.

## Author contributions

SB, EL, EJ, SC, and JL contributed to the design of the study. SB, EL, SC, and JL produced data. All authors contributed to the analysis of the data, to the redaction and/or the edition of the article.

### Conflict of interest statement

The authors declare that the research was conducted in the absence of any commercial or financial relationships that could be construed as a potential conflict of interest.
