# Supplementary material for: Antimicrobial Susceptibility among Urban Wastewater and Wild Shellfish Isolates of Non-O1/Non-O139 Vibrio cholerae from La Rance Estuary (Brittany, France)
Source: Front Microbiol. 2017 Sep 12;8:1637. doi: 10.3389/fmicb.2017.01637 (PMC5601046; doi:10.3389/fmicb.2017.01637)
Supplement: Supplementary file 1 [file Table1.docx]

Supplementary data

Table 1: Interpretative criteria used to determine antimicrobial susceptibility with the disk diffusion test in *Vibrio cholerae* isolates.

| Antimicrobial class | Antimicrobial agent | Disk content (µg) | Zone diameter interpretative criteria (mm) | | | Reference |
| --- | --- | --- | --- | --- | --- | --- |
|  |  |  | Susceptible | Intermediate | Resistant |  |
| ß-lactams | Ampicillin | 10 | ≥17 | 14-16 | ≤13 | (CLSI, 2015) |
|  | Amoxicillin-clavulanic acid | 20/10 | ≥18 | 14-17 | ≤13 | (CLSI, 2015) |
|  | Cefotaxime | 30 | ≥26 | 23-25 | ≤22 | (CLSI, 2015) |
|  | Imipenem | 10 | ≥23 | 20-22 | ≤19 | (CLSI, 2015) |
| Phenicols | Chloramphenicol | 30 | ≥18 | 13-17 | ≤12 | (CLSI, 2015) |
| Aminoglycosides | Amikacin | 30 | ≥17 | 15-16 | ≤14 | (CLSI, 2015) |
|  | Gentamicin | 10 | ≥15 | 13-14 | ≤12 | (CLSI, 2015) |
|  | Streptomycin | 10 | ≥15 | 12-14 | ≤11 | (CLSI, 2016) |
| (Fluoro)quinolones | Ciprofloxacin | 5 | ≥21 | 16-20 | ≤15 | (CLSI, 2015) |
|  | Nalidixic acid | 30 | ≥19 | 14-18 | ≤13 | (CLSI, 2016) |
|  | Norfloxacin | 10 | ≥17 | 13-16 | ≤12 | (CLSI, 2016) |
| Folate pathway inhibitors | Sulfonamides | 300 | ≥17 | 13-16 | ≤12 | (CLSI, 2015) |
|  | Trimethoprim-sulfamethoxazole | 1.25/23.75 | ≥16 | 11-15 | ≤10 | (CLSI, 2015) |
| Tetracyclines | Tetracycline | 30 | ≥15 | 12-14 | ≤11 | (CLSI, 2015) |
|  | Doxycycline | 30 | - | - | - |  |
| Macrolides | Erythromycin | 15 | - | - | - |  |

Interpretative criteria specific for *Vibrio* *spp*., including *V. cholerae* described in CLSI document M45 3^rd^ edition (CLSI, 2015), are adapted from those for *Enterobacteriaceae* M100 25S (CLSI, 2010). In grey, streptomycin, nalidixic acid, norfloxacin , antimicrobial agents for whose no breakpoint is available in M45 3^rd^ edition (CLSI, 2015), so breakpoints described for *Enterobacteriaceae* in M100 26S (CLSI, 2016) were used, as done in previous studies (Bier et al., 2015; Ceccarelli et al., 2015). For doxycycline and erythromycin (in blue), no breakpoint is available
